# Supplementary material for: Automatic identification and morphological comparison of bivalve and brachiopod fossils based on deep learning
Source: PeerJ. 2023 Oct 11;11:e16200. doi: 10.7717/peerj.16200 (PMC10576495; doi:10.7717/peerj.16200)
Supplement: Appendix S1 — (Punyasena et al., 2012; Kong, Punyasena & Fowlkes, 2016; Solano, Gasmen & Marquez, 2018; Dionisio et al., 2020; Hou et al., 2020; Liu & Song, 2020; Pires De Lima et al., 2020; Zhang et al., 2020; Foxon, 2021; Lallensack, Romilio & Falkingham, 2022; Niu & Xu, 2022; Wang et al., 2022; Ho et al., 2023; Hou et al., 2023); Liu et al. 2023; (Wang et al., 2023). [file peerj-11-16200-s001.docx]

**Appendix S1.** Details of the relevant studies (Punyasena et al. 2012; Kong et al. 2016; Solano et al. 2018; Dionisio et al. 2020; Hou et al. 2020; Liu and Song 2020; Pires De Lima et al. 2020; Zhang et al. 2020; Foxon 2021; Lallensack et al. 2022; Niu and Xu 2022; Wang et al. 2022; Ho et al. 2023; Hou et al. 2023; Liu et al. 2023; Wang et al. 2023).

| Author | Year | Category | The amount of taxa | Accuracy | Note |
| --- | --- | --- | --- | --- | --- |
| Lallensack et al. | 2022 | Fossil Tracks | 2 orders | 0.86 | Dinosaur tracks |
| Punyasena et al. | 2012 | Fossil Pollen | 2 species | 0.94 | Microfossil, slide-level analysis |
| Zhang et al. | 2020 | Tubular Fossils, Spherical Fossils, and Dross | 3 groups | >0.84 | This paper only shows the accuracy for each category |
| Kong et al. | 2016 | Fossil Pollen | 3 species | 0.86 | Using confocal fluorescence microscopy |
| Solano et al. | 2018 | Radiolarian | 4 species | 0.89 | / |
| Wang et al. | 2022 | Brachiopod | 5 species | 0.97 | Based on Transpose Convolutional Neural Network |
| Ho et al. | 2023 | Calcareous Algae, Mollusca, and Anthozoa | 6 groups | 0.97 | Carbonate skeletal grains |
| Pires De Lima  et al. | 2020 | Fusulinids | 8 genera | 0.89 | / |
| Dionisio et al. | 2020 | Radiolarian | 9 genera | 0.92 | Using scanning electron micrographs |
| Wang et al. | 2023 | Microfossils | 9 groups | 0.97 | / |
| Hou et al. | 2020 | Fish Scales | 11 groups | 0.98 | 3D digital microfossil |
| Foxon | 2021 | Ammonoid | 11 species | 0.78 | Using measurement data |
| Hou et al. | 2023 | Fusulinid | 16 genera | 0.92 | Preprint |
| Liu and Song | 2020 | Fossils and Abiotic Grains | 22 groups | 0.95 | Carbonate microfacies |
| Liu et al. | 2022 | Invertebrates, Vertebrates, Plants, Microfossils, and Trace Fossil | 50 clades | 0.90 | Using web crawlers |
| Niu and Xu | 2022 | Graptolite | 113 species | 0.71 | Preprint |
