# Supplementary material for: Automatic identification and morphological comparison of bivalve and brachiopod fossils based on deep learning
Source: PeerJ. 2023 Oct 11;11:e16200. doi: 10.7717/peerj.16200 (PMC10576495; doi:10.7717/peerj.16200)
Supplement: Appendix S2 — Every square represents a publication, and the numbers represent the number of images from each publication. [file peerj-11-16200-s002.pdf]

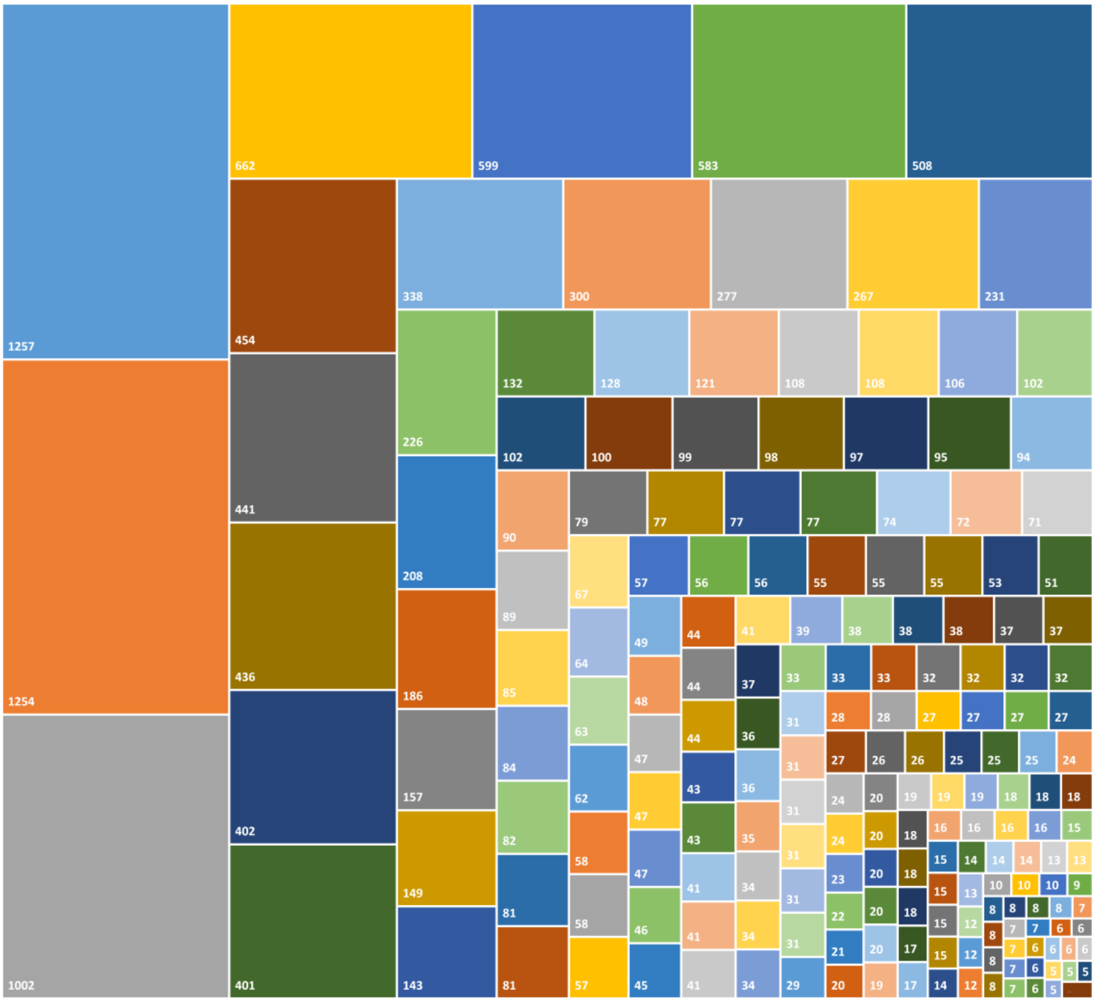

Appendix S2. The contribution of each publication to the dataset. Every square represents a publication, and the numbers represent the number of images from each publication.
