# Supplementary material for: Automatic identification and morphological comparison of bivalve and brachiopod fossils based on deep learning
Source: PeerJ. 2023 Oct 11;11:e16200. doi: 10.7717/peerj.16200 (PMC10576495; doi:10.7717/peerj.16200)
Supplement: Appendix S4 — The two correspond well to the logarithmic relationship. [file peerj-11-16200-s004.pdf]

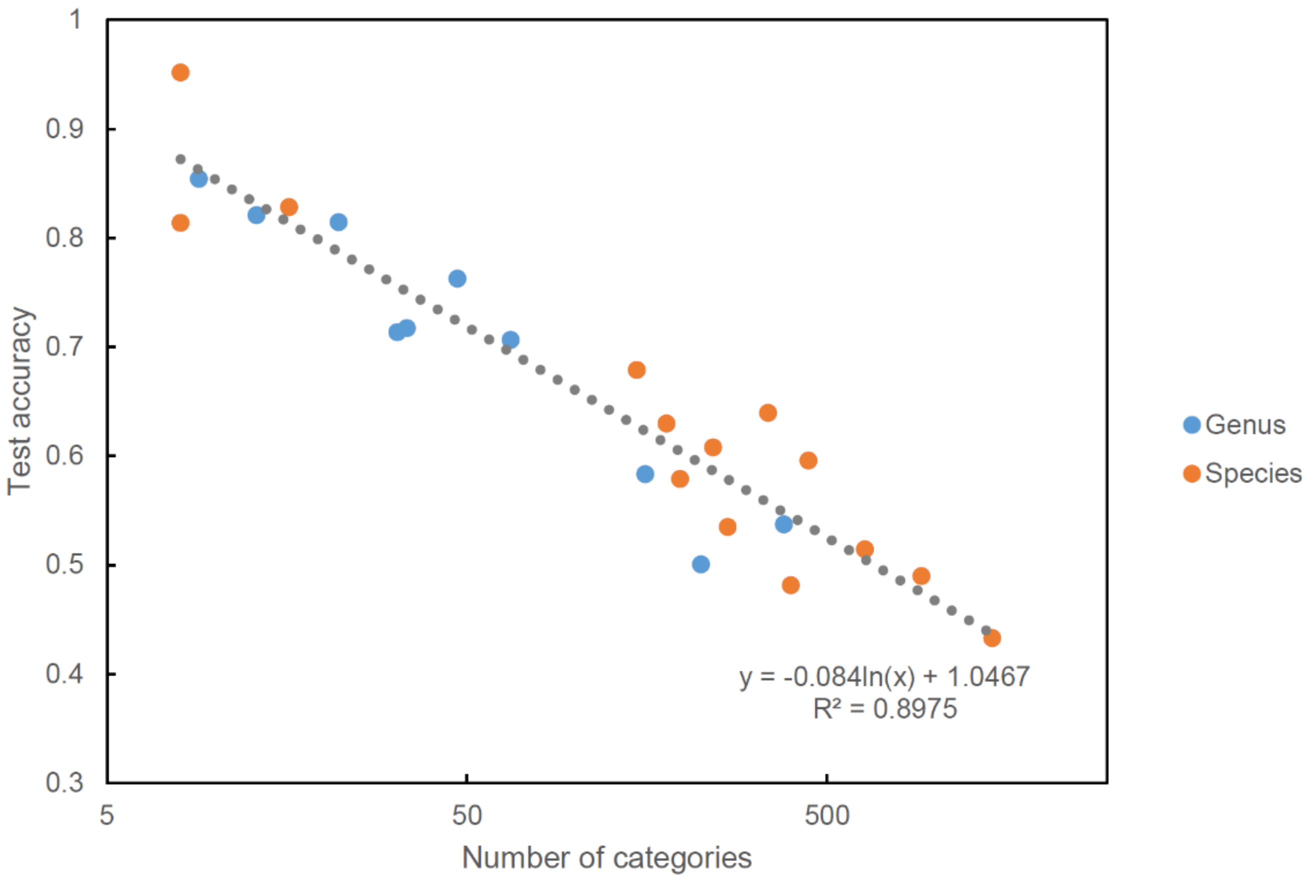

Appendix S4. Relationship between the number of categories and the accuracy. The two correspond well to the logarithmic relationship.
