# Supplementary material for: Automatic identification and morphological comparison of bivalve and brachiopod fossils based on deep learning
Source: PeerJ. 2023 Oct 11;11:e16200. doi: 10.7717/peerj.16200 (PMC10576495; doi:10.7717/peerj.16200)
Supplement: Appendix S5 — These images are all from the test set, where the orange border represents the bivalves and the blue border represents the brachiopods. Fossil images are not to scale. [file peerj-11-16200-s005.pdf]

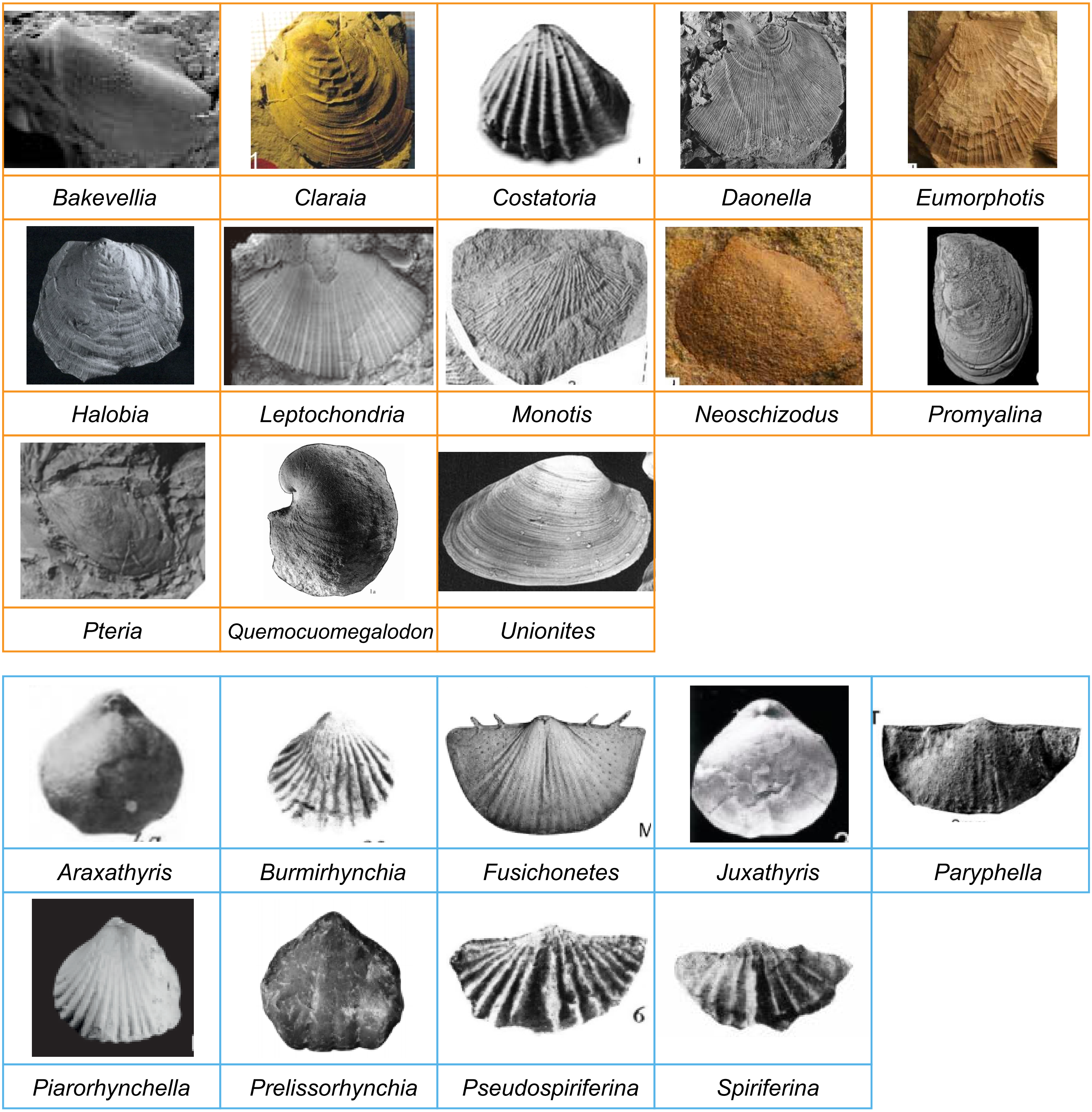

Appendix S5. Example images of all 22 genera in the scenario of Order 22. These images are all from the test set, where the orange border represents the bivalves and the blue border represents the brachiopods. Fossil images are not to scale.
