# Supplementary material for: Automatic identification and morphological comparison of bivalve and brachiopod fossils based on deep learning
Source: PeerJ. 2023 Oct 11;11:e16200. doi: 10.7717/peerj.16200 (PMC10576495; doi:10.7717/peerj.16200)
Supplement: Appendix S6 — The accuracy of the original identification model is 59.57%. The horizontal and vertical coordinates in the figure are the two dimensions obtained by t-SNE. The numbers represent different species. [file peerj-11-16200-s006.pdf]

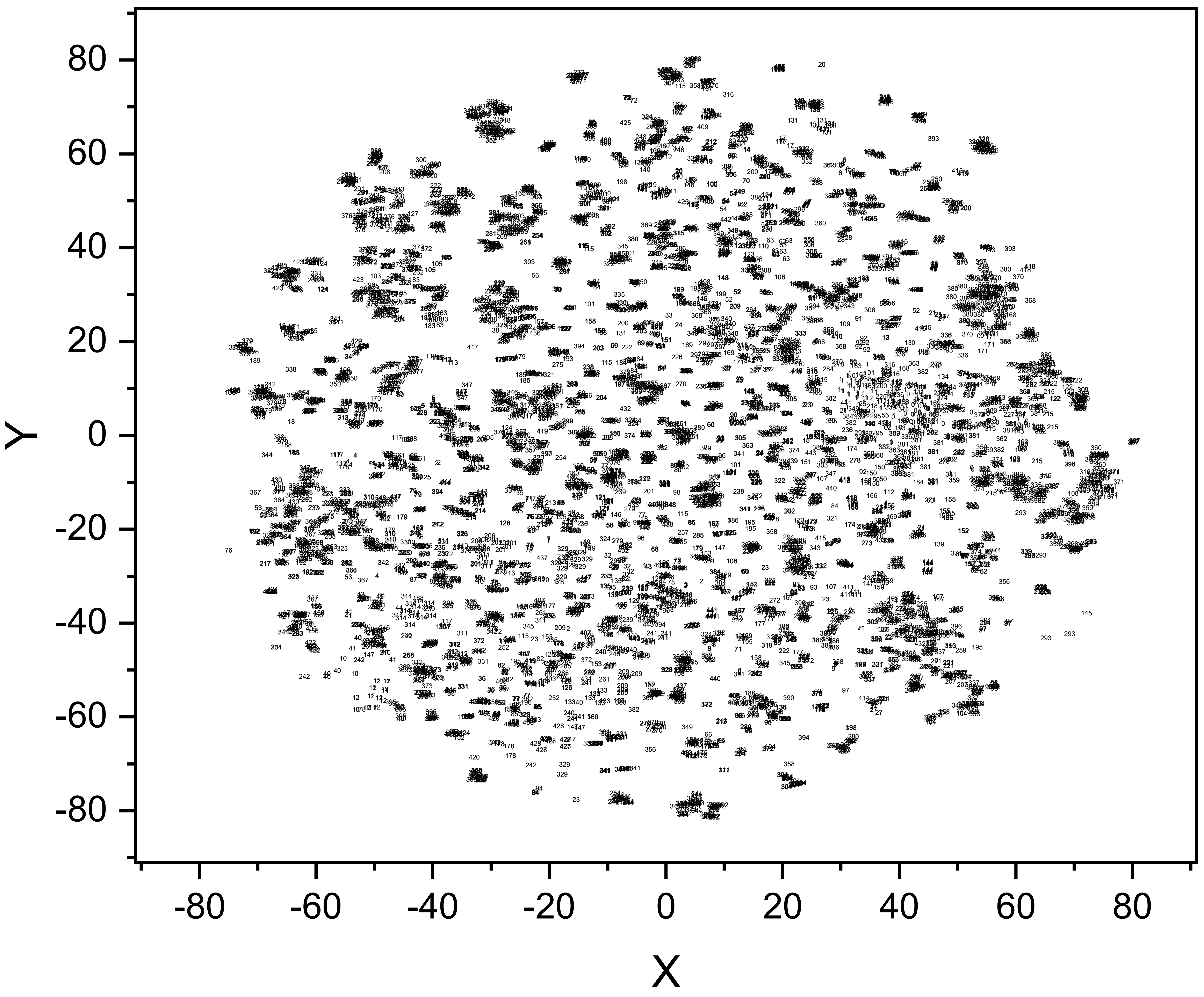

Appendix. Fossil morphological feature distribution maps for more categories (444 categories,8536 fossil images, based on Order 34) calculated by t-SNE. The accuracy of the original identification model is 59.57%. The horizontal and vertical coordinates in the figure are the two dimensions obtained by t-SNE. The numbers represent different species.
