# Supplementary material for: Automatic identification and morphological comparison of bivalve and brachiopod fossils based on deep learning
Source: PeerJ. 2023 Oct 11;11:e16200. doi: 10.7717/peerj.16200 (PMC10576495; doi:10.7717/peerj.16200)
Supplement: Supplemental Information 9 [file peerj-11-16200-s009.docx]

# Appendix-References for image dataset

Baeza-Carratala, J.F., Perez-Valera, F. and Perez-Valera, J.A., 2018. The oldest post-Paleozoic (Ladinian, Triassic) brachiopods from the Betic Range, SE Spain. Acta Palaeontologica Polonica, 63(1), 71-85.

Bakke, N., 2017. The evolution of the Triassic bivalve *Daonella* into *Halobia* in the Botneheia Formation on Svalbard-a sedimentological and palaeoenvironmental interpretation, Master's thesis, NTNU.

Balini, M. et al., 2015. The Carnian/Norian boundary succession at Berlin-Ichthyosaur State Park (Upper Triassic, central Nevada, USA). Paläontologische Zeitschrift, 89(3), 399-433.

Begg, J. and Campbell, H., 1985. *Etalia*, a new Middle Triassic (Anisian) bivalve from New Zealand, and its relationship with other pteriomorphs. New Zealand Journal of Geology and Geophysics, 28(4), 725-741.

Benatov, S., 2000. Brachiopod biostratigraphy of the Middle Triassic in Bulgaria and comparison with elsewhere in Europe, 4^th^ International Brachiopod Congress. Systematics Association Special Volume Series. Taylor & Francis Ltd, Nat Hist Museum, London, England, pp. 384-393.

Benatov, S.n., 1997. *Costirhynchopsis vidlicis* (Urosevic, 1981)-a new brachiopod species for the Bulgarian Middle Triassic. Geologica Balcanica, 27, 37-44.

Biakov, A., 2011. New species of Inoceramus-like bivalves of the genus *Aphanaia Koninck* from the Lower Permian of northeast Asia. Paleontological Journal, 45(1), 5-12.

Biakov, A., 2012. New Inoceramus-like bivalves of the genus *Kolymia* Licharew from the Middle Permian of Northeast Asia. Paleontological Journal, 46(6), 552-559.

Biakov, A., 2013. New species of the Inoceramus-like bivalve genus *Maitaia* Marwick from the Permian of northeastern Asia. Paleontological Journal, 47(5), 463-469.

Biakov, A. and Kutygin, R., 2018. Bivalves from the Delendzhian–Dulgalakhian boundary beds of the Middle Permian of the lower reaches of the Lena River (Northern Verkhoyansk Region, Northern Siberia). Paleontological Journal, 52(7), 761-767.

Bingde, S., 1982. Bivalvia. Palaeontological Atlas of Northwest China, Shaanxi Gansu Ningxia, 3, 18-20.

Bittner, A., 1890. Brachiopoden der alpinen Trias. Hölder.

Blodgett, R.B., Reifenstuhl, R. and Decker, P., 2004. Paleontology and stratigraphy of the Upper Triassic Kamishak Formation in the Puale Bay–Cape Kekurnoi–Alinchak Bay area, Karluk C-4 and C-5 quadrangle, Alaska Peninsula. Bristol Bay–Alaska Peninsula Region, Overview of, 2007, 131-160.

Boyd, D.W. and Newell, N.D., 2002. A unique pterioid bivalve from the Early Triassic of Utah. American Museum Novitates, 2002(3375), 1-9.

Bradshaw, M., 1984. Permian nonmarine bivalves from the Ohio Range, Antarctica. Alcheringa, 8(4), 305-309.

Broglio Loriga, C., Neri, C., Pasini, M. and Posenato, R., 1986. Marine fossil assemblages from Upper Permian to lowermost Triassic in the western Dolomites (Italy). Memorie della Società Geologica Italiana, 34, 5-44.

Broili, F., 1907. Die Fauna der Pachycardientuffe der Seiser Alp. Scaphopoden und Gastropoden. Palaeontographica (1846-1933). 69-138.

Campbell, J.D., 1987. Triassic records of the genus *Lingula* (Brachiopoda, Inarticulata) in New-Zealand. Journal of the Royal Society of New Zealand, 17(1), 9-16.

Chen, J.-H. and Stiller, F., 2007. The halobiid bivalve genus *Enteropleura* and a new species from the Middle Anisian of Guangxi, southern China. Acta Palaeontologica Polonica, 52(1), 53-61.

Chen, J.-h. and Stiller, F., 2010. An early *Daonella* from the Middle Anisian of Guangxi, southwestern China, and its phylogenetical significance. Swiss Journal of Geosciences, 103(3), 523-533.

Chen, J., Chen, Z.-Q. and Tong, J.-N., 2010. Palaeoecology and taphonomy of two brachiopod shell beds from the Anisian (Middle Triassic) of Guizhou, Southwest China: recovery of benthic communities from the end-Permian mass extinction. Global and Planetary Change, 73(1-2), 149-160.

Chen, J. and Yang, Z., 1989. Occurrence of Triassic bivalves in the Karakorum Mountain area. Xinjiang Geology.(2), 62-73.

Chen, Z.-Q., Kaiho, K., George, A.D. and Tong, J., 2006. Survival brachiopod faunas of the end-Permian mass extinction from the southern Alps (Italy) and South China. Geological Magazine, 143(3), 301-327.

Chen, Z. and Shi, G., 1999. Revision of Prelissorhynchia Xu and Grant, 1994 (Brachiopoda) from the Upper Permian of South China. Proceedings of the Royal Society of Victoria, 111(1), 15-26.

Chen, Z., Shi, G., Shu-Zhong, S. and Archbold, N., 2000. *Tethyochonetes* gen. nov.(Chonetida, Brachiopoda) from the Late Permian of China. 1-15.

Chen, Z., Shi, G.R. and Kaiho, K., 2002. A new genus of rhynchonellid brachiopod from the Lower Triassic of South China and implications for timing the recovery of Brachiopoda after the end‐Permian mass extinction. Palaeontology, 45(1), 149-164.

Chen, Z.Q. and Liao, Z.T., 2009. Brachiopod faunas across the Wuchiapingian-Changhsingian (Late Permian) boundary at the stratotype section and subsurface of Changxing area, South China. Neues Jahrbuch Fur Geologie Und Palaontologie-Abhandlungen, 254(3), 315-335.

Chen, Z.Q. et al., 2009. Environmental and biotic turnover across the Permian-Triassic boundary on a shallow carbonate platform in western Zhejiang, South China. Australian Journal of Earth Sciences, 56(6), 775-797.

Cox, L.R., 1924. II.—A Triassic Fauna from the Jordan Valley. Annals and Magazine of Natural History, 14(79), 52-96.

Cui, X. and Liu, B., 1987. Late triassic-early jurassic bivalve fauna from Zanda County in southwest Xizang(Tibet), China. Geoscience, 1(2), 196.

Dagys, A., 1965. Triassic brachiopods of Siberia. Nauka, Moskva.

Dagys, A., 1974. Triassic brachiopods (morphology, classification, phylogeny, stratigraphical significance and biogeography). Akademiia Nauk SSSR, Sibirskoe Otdelenie, Institut Geologii i Geofiziki, Trudy, 214, 386.

Dagys, A., 1985. The Triassic brachiopods and bivalves of the north of Central Siberia. Transactions of Geology Geophys. Siberian Branch, 633, 1-160.

Damborenea, S.E., Echevarría, J. and Ros-Franch, S., 2012. Southern hemisphere palaeobiogeography of Triassic-Jurassic marine bivalves. Springer Science & Business Media.

Damborenea, S.E. and González-León, C.M., 1997. Late Triassic and Early Jurassic bivalves from Sonora, Mexico. Revista Mexicana de Ciencias Geológicas, 14(2), 178-201.

De Toni, A., 1912. Brachiopodi della zona a ceratites trinodosus di Monte Rite in Cadora. Soc. Coop. Tipogr.

Del Piero, N., Rigaud, S., Takahashi, S., Poulton, S.W. and Martini, R., 2020. Unravelling the paleoecology of flat clams: New insights from an Upper Triassic halobiid bivalve. Global and Planetary Change, 190, 103195.

Di Stefano, P. et al., 2012. Middle Triassic (Ladinian) deep-water sediments in Sicily: new findings from the Madonie Mountains. Rivista Italiana di Paleontologia e Stratigrafia, 118(2), 235-246.

Feldman, H.R., 2002. A new species of *Coenothyris* (Brachiopoda) from the Triassic (Upper Anisian-Ladinian) of Israel. Journal of Paleontology, 76(1), 34-42.

Feldman, H.R., 2013. *Menathyris wilsoni* (Brachiopoda), new genus and species from the middle Triassic (Ladinian) of southern Israel. Annales Societatis Geologorum Poloniae, 83(2), 81-85.

Feldman, H.R., 2017. *Tunethyris blodgetti* sp nov (Brachiopoda, Terebratulida) from the middle Triassic of Makhtesh Ramon, southern Israel. Annales Societatis Geologorum Poloniae, 87(1), 89-99.

Feng, Q., Cui, X. and Liu, B., 1992. Discovery of Late Permian bivalve-fauna from Laochang of Lancang, West Yunnan and its biogeographic characteristics. Earth Science–Journal of China University of Geosciences, 17(5), 512-520.

Feng, S., Meng, F. and Chen, G., 1977. Atlas of paleontology in central and southern China.

Foster, W.J., Danise, S., Price, G.D. and Twitchett, R.J., 2017a. Subsequent biotic crises delayed marine recovery following the late Permian mass extinction event in northern Italy. Plos One, 12(3), 24.

Foster, W.J., Danise, S. and Twitchett, R.J., 2017b. A silicified Early Triassic marine assemblage from Svalbard. Journal of Systematic Palaeontology, 15(10), 851-877.

Freneix, S. and Avias, J., 1977. Caledogonia, a new smooth trigoniacean bivalve from the Triassic of New Caledonia. Alcheringa, 1(3), 279-291.

Fürsich, F., Hautmann, M., Senowbari-Daryan, B. and Seyed-Emami, K., 2005. The Upper Triassic Nayband and Darkuh formations of east-central Iran: Stratigraphy, facies patterns and biota of extensional basins on an accreted terrane. Beringeria, 35, 53-133.

Fürsich, F.T. and Hautmann, M., 2005. Bivalve reefs from the Upper Triassic of Iran. Sezione di Museologia Scientifica e Naturalistica, 1, 13-24.

Gaetani, M., 2016. Brachiopods from the type-section of the Bithynian substage (Anisian, Middle Triassic, Northwestern Turkey). Rivista italiana di Paleontologia e Stratigrafia, 122(2), 61-75.

Gaetani, M., Balini, M., Nicora, A., Giorgioni, M. and Pavia, G., 2018. The Himalayan connection of the Middle Triassic brachiopod fauna from Socotra (Yemen). Bulletin of Geosciences, 93(2), 247-268.

Gair, H., Gregg, D. and Speden, I., 1962. Triassic fossils from Corbies Creek, North Otago. New Zealand Journal of Geology and Geophysics, 5(1), 92-113.

Gao, Y., Shi, G. and Peng, Y., 2009. A new bivalve fauna from the Permian–Triassic boundary section of southwestern China. Alcheringa, 33(1), 33-47.

Geology, N.I.o. and Paleontology, 1976. Chinese fossils of all groups: Fossil lamellibranchia of China. Science Press.

Geyer, G., Hautmann, M., Hagdorn, H., Ockert, W. and Streng, M., 2005. Well-preserved mollusks from the lower Keuper (Ladinian) of Hohenlohe (Southwest Germany). Paläontologische Zeitschrift, 79(4), 429-460.

González, C.R., 2006. Lower permian bivalves from central Patagonia, Argentina. Paläontologische Zeitschrift, 80(2), 130-155.

Gou, Z., 1993. Bivalve fauna of upper Triassic in Maantang area, Jiangyou, Sichuan. Acta Palaeontologica Sinica, 32(1), 13-30.

Gou, Z., 1998. The bivalve faunas from the Upper Triassic Xujiahe Formation in the Sichuan basin. Lithofacies Paleogeography, 18(2), 20-31.

Gradinaru, E. and Gaetani, M., 2019. Upper Spathian to Bithynian (lower to middle Triassic) brachiopods from north Dobrogea (Romania). Rivista Italiana di Paleontologia e Stratigrafia, 125(1), 91-123.

Grant-Mackie, J., 1978. Status and identity of the New Zealand Upper Triassic bivalve *Monotis salinaria* var. richmondiana Zittel 1864. New Zealand Journal of Geology and Geophysics, 21(3), 375-402.

Grant-Mackie, J. and Silberling, N., 1990. New data on the Upper Triassic bivalve *Monotis* in North America, and the new subgenus *Pacimonotis*. Journal of Paleontology, 64(2), 240-254.

Grunt, T., 1986. Classification of brachiopods of the order Athyridida. Trudy Paleontologičeskogo Instituta Akademii Nauk SSSR, 215, 1-200.

Guo, Z., Chen, Z.Q. and Harper, D.A.T., 2020. The Anisian (Middle Triassic) brachiopod fauna from Qingyan, Guizhou, south-western China. Journal of Systematic Palaeontology, 18(8), 647-701.

Halamski, A.T., Bitner, M.A., Kaim, A., Kolar-Jurkovsek, T. and Jurkovsek, B., 2015. Unusual brachiopod fauna from the Middle Triassic algal meadows of Mt. Svilaja (Outer Dinarides, Croatia). Journal of Paleontology, 89(4), 553-575.

Hautmann, M., 2001. Die Muschelfauna der Nayband-Formation (Obertrias, Nor-Rhät) des östlichen Zentraliran. 1-181.

Hautmann, M., 2008. Taxonomy and phylogeny of the Triassic bivalve families Mysidiellidae Cox, 1964 and Healeyidae new family. Journal of Paleontology, 82(3), 555-564.

Hautmann, M., Aghababalou, B. and Krystyn, L., 2011. An unusual Late Triassic nuculid bivalve with divaricate shell ornamentation, and the evolutionary history of oblique ribs in Triassic bivalves. Journal of Paleontology, 85(1), 22-28.

Hautmann, M. and Hagdorn, H., 2013. Oysters and oyster-like bivalves from the Middle Triassic Muschelkalk of the Germanic Basin. Paläontologische Zeitschrift, 87(1), 19-32.

Hautmann, M. and Nützel, A., 2005. First record of a heterodont bivalve (Mollusca) from the Early Triassic: palaeoecological significance and implications for the ‘Lazarus problem’. Palaeontology, 48(6), 1131-1138.

Hautmann, M., Smith, A.B., McGowan, A.J. and Bucher, H., 2013. Bivalves from the Olenekian (Early Triassic) of south-western Utah: systematics and evolutionary significance. Journal of Systematic Palaeontology, 11(3), 263-293.

Hayami, I., Maeda, S. and Fuller, C.R., 1977. 683. Some late Triassic bivalvia and gastropoda from the domeyko range of north Chile, Transactions and proceedings of the Paleontological Society of Japan. New series. Palaeontological Society of Japan, pp. 202-221.

He, W. et al., 2007. A late Permian to early Triassic bivalve fauna from the Dongpan Section, southern Guangxi, South China. Journal of Paleontology, 81(5), 1009-1019.

He, W. et al., 2015. A new genus *Liaous* of early Anisian Stage (Middle Triassic) brachiopods from southwestern China: systematics, reassessment of classification of the Spiriferinioidea, community paleoecology, and paleoenvironmental implications. Journal of Paleontology, 89(6), 966-979.

He, W.H. et al., 2014. Changhsingian (latest Permian) deep-water brachiopod fauna from South China. Journal of Systematic Palaeontology, 12(8), 907-960.

Hofmann, R. et al., 2014. Recovery of benthic marine communities from the end-Permian mass extinction at the low latitudes of eastern P anthalassa. Palaeontology, 57(3), 547-589.

Hofmann, R., Hautmann, M. and Bucher, H., 2013a. A new paleoecological look at the Dinwoody Formation (Lower Triassic, Western USA): intrinsic versus extrinsic controls on ecosystem recovery after the end-Permian mass extinction. Journal of Paleontology, 87(5), 854-880.

Hofmann, R., Hautmann, M. and Bucher, H., 2015. Recovery dynamics of benthic marine communities from the Lower Triassic Werfen Formation, northern Italy. Lethaia, 48(4), 474-496.

Hofmann, R., Hautmann, M., Wasmer, M. and Bucher, H., 2013b. Palaeoecology of the Spathian Virgin Formation (Utah, USA) and its implications for the Early Triassic recovery. Acta Palaeontologica Polonica, 58(1), 149-173.

Hoover, P.R., 1980. Early Triassic terebratulid brachiopods from the western interior of the United States. Early Triassic Terebratulid Brachiopods from the Western Interior of the United States.(P-1057).

Hopkin, E.K. and McRoberts, C.A., 2005. A new Middle Triassic flat clam (Pterioida: Halobiidae) from the Middle Anisian of north-central Nevada, USA. Journal of Paleontology, 79(4), 796-800.

Hornung, T., Krystyn, L. and Brandner, R., 2007. A Tethys-wide mid-Carnian (Upper Triassic) carbonate productivity crisis: evidence for the Alpine Reingraben event from Spiti (Indian Himalaya)? Journal of Asian Earth Sciences, 30(2), 285-302.

Huang, Y., 2014. Extinction and recovery of bivalves during the Permian–Triassic Transition. China University of Geosciences. 155.

Huang, Y., Tong, J. and Fraiser, M.L., 2018. A Griesbachian (Early Triassic) mollusc fauna from the Sidazhai Section, Southwest China, with paleoecological insights on the proliferation of genus *Claraia* (Bivalvia). Journal of Earth Science, 29(4), 794-805.

Ichikawa, K. and Yin, E., 1966. Discovery of Early Triassic bivalves from Kelantan, Malaya: Notes on. Journal of geosciences Osaka City University, 9, 101-108.

Iordan, M., 1993. Triassic brachiopods of Romania. Mesozoic brachiopods of Alpine Europe. Hungarian Geological Society, Budapest. 49-54.

Jin-hua, C. and Chu-zhen, C., 1980. *Jianchuania*, a new Genus of Bivalve from the Upper Triassic in Yunnan. Acta Palaeontologica Sinica, 19(1), 57-60.

Kashiyama, Y. and Oji, T., 2004. Low-diversity shallow marine benthic fauna from the Smithian of northeast Japan: paleoecologic and paleobiogeographic implications. Paleontological Research, 8(3), 199-218.

Kolar-Jurkovsek, T. et al., 2013. Olenekian (Early Triassic) fossil assemblage from eastern Julian Alps (Slovenia), Annales Societatis Geologorum Poloniae. Geological Society of Poland, 213-227.

Komatsu, T., Akasaki, M., Chen, J.-H., Cao, M.-Z. and Stiller, F., 2004a. Benthic fossil assemblages and depositional facies of the Middle Triassic (Anisian) Yuqing Member of the Qingyan Formation, southern China. Paleontological Research, 8(1), 43-52.

Komatsu, T., Chen, J.-h., Cao, M.-z., Stiller, F. and Naruse, H., 2004b. Middle Triassic (Anisian) diversified bivalves: depositional environments and bivalve assemblages in the Leidapo Member of the Qingyan Formation, southern China. Palaeogeography, Palaeoclimatology, Palaeoecology, 208(3-4), 207-223.

Komatsu, T. and Huyen, D.T., 2007. Lower Triassic bivalve fossils from the Song Da and An Chau Basins, North Vietnam. Paleontological Research, 11(2), 135-144.

Komatsu, T., Huyen, D.T. and Huu, N.D., 2010. Radiation of Middle Triassic bivalve: Bivalve assemblages characterized by infaunal and semi-infaunal burrowers in a storm-and wave-dominated shelf, An Chau Basin, North Vietnam. Palaeogeography, Palaeoclimatology, Palaeoecology, 291(3-4), 190-204.

Komatsu, T., Huyen, D.T. and Jin-Hua, C., 2008. Lower Triassic bivalve assemblages after the end-Permian mass extinction in South China and North Vietnam. Paleontological Research, 12(2), 119-128.

Komatsu, T. et al., 2013. *Crittendenia* (Bivalvia) from the Lower Triassic (Olenekian) Bac Thuy Formation, An Chau Basin, Northern Vietnam. Paleontological Research, 17(1), 1-11.

Komatsu, T., Tran, H.D. and Chen, J.-H., 2006. Depositional environments and fossil bivalves in the lowermost parts of the Triassic systems in North Vietnam and south China. Journal of Geography (Chigaku Zasshi), 115(4), 470-483.

Kummel, B., 1970. Stratigraphic boundary problems: Permian and Triassic of West Pakistan. Edited by Bernhard Kummel and Curt Teichert. University of Kansas Press.

Larghi, C., Balini, M. and Torti, V., 2006. A new *Daonella* from the Ladinian platform of the Esino limestone (Southern alps, Italy). Rivista Italiana di Paleontologia e Stratigrafia, 112(1), 167-174.

Liao, Z.-t., 1984. New genus and species of Late Permian and earliest Triassic brachiopods from Jiangsu, Zhejiang and Anhui Provinces. Acta Palaeontologica Sinica, 23, 276-284.

Lieberman, H., 1979. Die bivalen-und ostracodenfauna von raibl und ihr stratigraphischer wert.

Lin, L. et al., 2011. Late Triassic bivalves associated with a hydrothermal vent system in the Yidun Island Arc (SW China) of the eastern Tethys. Science China Earth Sciences, 54(12), 1864-1870.

Ling, Q., 1988. Triassic bivalve fauna in Zhen'an Area of east Qinling Mts. Acta Palaeontologica Sinica, 5, 615-628.

Liu, S., 2017. Early Triassic sedimentray environmental evolution and its biological responses in the Xingyi region, Guizhou. Chengdu University of Technology. 1-140.

MacFarlan, D.A.B. and Campbell, J., 1992. Triassic and Jurassic *Rhynchonellacea* (Brachiopoda) from New Zealand & New Caledonia. Royal Society of New Zealand.

Malinowska, L., 1986. Geology of Poland. III Atlas of guide and characteristic fossils. 2a Mesozoic. Triassic.–353.

Mansfield, G.R., 1927. Geography, geology, and mineral resources of part of southeastern Idaho, 152. US Government Printing Office.

Mantovani, N., 2002. The genus *Tetractinella* Bittner, 1890: Morphology, ultrastructure, and 3D reconstruction. Rivista Italiana Di Paleontologia E Stratigrafia, 108(1), 37-50.

McRoberts, C.A., 1990. Systematic paleontology stratigraphic occurrence and paleoecology of halobiid bivalves from the Martin Bridge Formation (Upper Triassic) Wallowa Terrane Oregon.

McRoberts, C.A., 1992. Systematics and paleobiogeography of Late Triassic Gryphaea (Bivalvia) from the North American Cordillera. Journal of Paleontology, 66(1), 28-39.

McRoberts, C.A., 1993. Systematics and biostratigraphy of halobiid bivalves from the Martin Bridge Formation (Upper Triassic), northeast Oregon. Journal of Paleontology, 67(2), 198-210.

McRoberts, C.A., 1997. Late Triassic (Norian-Rhaetian) bivalves from the Antimonio Formation, northwestem Sonora, Mexico. Revista Mexicana de Ciencias Geológicas, 14(2), 167-177.

McRoberts, C.A., 2000. A primitive *Halobia* (Bivalvia: Halobioidea) from the Triassic of northeast British Columbia. Journal of Paleontology, 74(4), 599-603.

McRoberts, C.A., 2010. Biochronology of Triassic bivalves. Geological Society, London, Special Publications, 334(1), 201-219.

McRoberts, C.A., 2011. Late Triassic bivalvia (Chiefly Halobiidae and Monotidae) from the Pardonet Formation, Williston Lake Area, Northeastern British Columbia, Canada. Journal of Paleontology, 85(4), 613-664.

McRobERts, C.A., 2017. A silicified late Triassic (Norian) bivalve molluscan fauna from the Alexander Terrane, southeastern Alaska. Bulletins of American Paleontology.(391), 1-95.

McRoberts, C.A. and Blodgett, R.B., 2000. Late Triassic (Norian) mollusks from the Taylor Mountains Quadrangle, southwestern Alaska. US Geological Survey Professional Paper, 1662, 55-67.

Michalík, J. et al., 2013. Paleoenvironments during the Rhaetian transgression and the colonization history of marine biota in the Fatric Unit (Western Carpathians). Geologica Carpathica, 64(1), 39-62.

Nakazawa, K. and Newell, D.N., 1968. Permian bivalves of Japan. Memoirs of the Faculty of Science, Kyoto University. Series of Geology and Mineralogy, 35(1), 1-108.

Neri, C. and Posenato, R., 1985. New biostratigraphical data on uppermost Werfen Formation of western Dolomites (Trento, Italy). Geologisch− Paläontologische Mitteilungen Innsbruck, 14, 83-107.

Newell, N.D. and Boyd, D.W., 1995. Pectinoid bivalves of the Permian-Triassic crisis. Bulletin of the AMNH; no. 227.

Newell, N.D. and Boyd, D.W., 1999. A new Lower Triassic *Permophorus* from the central Rocky Mountains. American Museum novitates; no. 3263.

Newell, N.D. and Kummel, B., 1942. Lower Eo-triassic stratigraphy, western Wyoming and southeast Idaho. Bulletin of the geological Society of America, 53(6), 937-996.

Niu, Y., Jiang, B. and Zhang, Y., 2013. The Triassic and early Jurassic bivaalve fossils from eastern south China and their biostratigraphic implications. Acta Palaeontologica Sinica, 52(3), 352-376.

Onoue, T. and Tanaka, H., 2005. Late Triassic bivalves from Sambosan accretionary complex, southwest Japan, and their biogeographic implications. Paleontological Research, 9(1), 15-25.

Pálfy, J., 1988. Middle Triassic rhynchonellids from the Balaton Highland (Transdanubian Central Range, Hungary). Annales Historico-Naturale Musei Nationalis Hungarici, 80, 25-46.

Pan, Y.H. et al., 2014. Early Triassic bivalves from the Feixianguan Formation in Xingyi, Guizhou and the Ximatang Formation in Qiubei, Yunnan (southern China). Palaeoworld, 23(2), 143-154.

Peng, Y.Q. and Shi, G.R., 2008. New Early Triassic Lingulidae (Brachiopoda) genera and species from South China. Alcheringa, 32(2), 149-170.

Perry, D. and Chatterton, B., 1979. Late Early Triassic brachiopod and conodont fauna, Thaynes Formation, southeastern Idaho. Journal of Paleontology. 307-319.

Popov, A.M. and Zakharov, Y.D., 2017. Olenekian Brachiopods from the Kamenushka River Basin, South Primorye: New data on the brachiopod recovery after the End-Permian mass extinction. Paleontological Journal, 51(7), 735-745.

Posenato, R., 2008a. Anisian (Middle Triassic) bivalves from the Dolomites (Italy). Neues Jahrbuch für Geologie und Paläontologie-Abhandlungen. 93-115.

Posenato, R., 2008b. Patterns of bivalve biodiversity from Early to Middle Triassic in the Southern Alps (Italy): regional vs. global events. Palaeogeography, Palaeoclimatology, Palaeoecology, 261(1-2), 145-159.

Posenato, R., Holmer, L.E. and Prinoth, H., 2014. Adaptive strategies and environmental significance of lingulid brachiopods across the late Permian extinction. Palaeogeography Palaeoclimatology Palaeoecology, 399, 373-384.

Posenato, R., Sciunnach, D. and Garzanti, E., 1996. First report of *Claraia* (bivalvia) in the servino formation (Lower Triassic) of the western Orobic Alps, Italy. Rivista Italiana di Paleontologia e Stratigrafia, 102(2), 201-210.

Rowell, A., 1970. *Lingula* from the basal Triassic Kathwai member, Mianwali formation, salt range and surghar range, West Pakistan. Stratigraphic Boundary Problems: Permian and Triassic of West Pakistan, 4, 111-116.

Scholz, G., 1972. An Anisian Wetterstein limestone reef in north Hungary. Acta Mineralogica-Petrographica, 20(2), 337-362.

Scientific Expedition Team to Xizang, C.A.o.S., 1976. Report of the scientific expedition in the Mount Qomolangma region, Paleontology, 2. Beijing: Science Press.

Senkowiczowa, H. and Popiel-Barczyk, E., 1996. Some *Terebratulida* (Brachiopoda) from the Muschelkalk sediments in the Holy Cross Mts. Geological Quarterly, 40(3), 443-466.

Shen, S.-z. and He, X.-l., 1994. Changhsingian brachiopod fauna from Guiding, Guizhou. Acta Palaeontologica Sinica, 33(4), 440-454.

Shen, S. and Archbold, N.W., 2001. *Chonetoidea* (Brachiopoda) from the Lopingian (Late Permian) of South China. Alcheringa, 25(3), 327-349.

Shen, S.Z., Grunt, T.A. and Jin, Y.G., 2004. A comparative study of Comelicaniidae Merla, 1930 (Brachiopoda : Athyridida) from the lopingian (late Permian) of South China and Transcaucasia in Azerbaijan and Iran. Journal of Paleontology, 78(5), 884-899.

Shen, S.Z., He, X.L. and Shi, G.G., 1995. Biostratigraphy and correlation of several Permian-Triassic boundary sections in southwestern China. Journal of Southeast Asian Earth Sciences, 12(1-2), 19-30.

Shigeta, Y., Maeda, H., Hakubutsukan, K.K. and Tokyo, J., 2009. The lower Triassic system in the Abrek Bay Area, South Primorye, Russia. 1-218.

Siblik, M., 1991. Triassic brachiopods from Aghdarband (NE-Iran). Abhandlungen der Geologischen Bundesanstalt, 38, 165-175.

Silantiev, V., Urazaeva, M. and Golubev, V., 2018. The nonmarine bivalve *Permianaia* Gen. Nov., the last member of Naiaditidae from the terminal Permian the east European Platform. Paleontological Journal, 52(7), 777-790.

Silantiev, V.V. and Urazaeva, M.N., 2017. *Hypoanthraconaia*: a new genus of non-marine bivalve molluscs from the Early Permian of Far East Russia. PalZ, 91(1), 71-84.

Silberling, N.J., 1963. Field guide to halobiid and monotid pelecypods of the Alaskan Triassic. US Geological Survey Open File Report, 1963, 1-9.

Silberling, N.J., Grant-Mackie, J.A. and Nichols, K.M., 1997. The Late Triassic bivalve *Monotis* in accreted terranes of Alaska. US Government Printing Office.

Simões, M.G. et al., 2012. Permian non-marine bivalves of the Falkland Islands and their palaeoenvironmental significance. Alcheringa: An Australasian Journal of Palaeontology, 36(4), 543-554.

Song, T., 2018. Study on the bivalve faunas in southwestern China during the Permian-Triassic transitional time. China University of Geosciences. 1-182.

Stiller, F. and Jinhua, C., 2004. *Eophilobryoidella sinoanisica* new genus and species, an early philobryid bivalve from the Upper Anisian (Middle Triassic) of Qingyan, southwestern China. Journal of Paleontology, 78(2), 414-419.

Sun, D. and Ye, S., 1982. Middle Triassic brachiopods from the Tosu Lake area, central Qinghai. Acta Palaeontologica Sinica, 21(2), 153-173.

Sun, Z., Hao, W., Sun, Y. and Jiang, D., 2009. Silicified Anisian (Middle Triassic) spiriferinid brachiopods from Guizhou, South China. Acta Palaeontologica Polonica, 54(1), 61-68.

Tamura, M. and McRoberts, C., 1993. A new species of *Myophorigonia* from the Upper Triassic Martin Bridge Formation, Wallowa Mountains, Oregon, USA with reference on the Minetrigoniidae of the Circum-Pacific. Minutes of the Division of Education, Kumamoto University Natural Sciences.(42), 29-34.

Tang, C. et al., 2007. Preliminary discussion on bivalves assemblages and their environments of the BagongFormation of upper triassic in Geladandongarea Yangtze Source Region. Journal of Palaeogeography, 9(1), 59-68.

Urazaeva, M., 2018. Revision of Late Permian nonmarine bivalves of the Genus *Opokiella* Plotnikov, 1949. Paleontological Journal, 52(7), 768-776.

Urlichs, M., 2011. Stunting in invertebrates from the type area of the Cassian Formation (Early Carnian) of the dolomites (Italy). GeoAlp, 8, 164-169.

Vörös, A., 1981. A survey of the Rhaetian (Upper Triassic) Bivalvia from Borzavár (Bakony Mts., Hungary), Annales Historico-Naturales Musei Nationalis Hungarici, pp. 33-54.

Vörös, A. and Budai, T., 2003. The Pelsonian substage on the Balaton Highland (Middle Triassic, Hungary). Institutum Geologicum Hungaricum.

Waller, T.R. and Stanley Jr, G.D., 2005. Middle Triassic pteriomorphian Bivalvia (mollusca) from the New Pass Range, west-central Nevada: systematics, biostratigraphy, paleoecology, and paleobiogeography. Memoir (The Paleontological Society). 1-64.

Wang, F.Y., Chen, J., Dai, X. and Song, H.J., 2017. A new dienerian (Early Triassic) brachiopod fauna from south China and implications for biotic recovery after the Permian-Triassic extinction. Papers in Palaeontology, 3(3), 425-439.

Wang, M.-Q., 1993. Bivalve fauna from uppermost Permian and lowermost Triassic of Fenghai, Yong'an, Fujian. Acta Palaeontologica Sinica, 32(4), 458-476.

Wang, Y., Zheng, Z. and Chen, G., 1979. Cephalopoda. Paleontological Atlas of Northwest China, Qinghai. 3-59.

Wasmer, M. et al., 2012. Olenekian (Early Triassic) bivalves from the Salt Range and Surghar Range, Pakistan. Palaeontology, 55(5), 1043-1073.

Waterhouse, J., 1960. Some Carnian pelecypods from New Zealand, Transactions of the Royal Society of New Zealand. The Society, pp. 425-442.

Waterhouse, J., 1980a. A new bivalve species (Buchiidae) from the Early Triassic of New Zealand. Alcheringa, 4(1), 1-10.

Waterhouse, J., 1980b. Permian bivalves from New Zealand. Journal of the Royal Society of New Zealand, 10(1), 97-133.

Wen, H., Shi, H. and Wang, D., 2011. New information and geological sense of bivalve fossils from the Waigucun Formation in southeast of the Simao basin, Yunnan, China. Geological Bulletin of China, 30(1), 179-183.

Westermann, G., 1966. New occurrences of *Monotis* from Canada (Triassic Pelecypoda). Canadian Journal of Earth Sciences, 3(7), 975-986.

Wu, F., 1985. New material of bivalves from the early Triassic in Fujian. Acta Palaeontologica Sinica, 24, 395-402.

Wu, H. et al., 2018. A new Permian–Triassic boundary brachiopod fauna from the Xinmin section, southwestern Guizhou, south China and its extinction patterns. Alcheringa: An Australasian Journal of Palaeontology, 42(3), 339-372.

Wu, H.T., Zhang, Y. and Sun, Y.L., 2019. A mixed Permian-Triassic boundary brachiopod fauna from Guizhou province, south China. Rivista Italiana di Paleontologia e Stratigrafia, 125(3), 609-630.

Wu, N., Wu, W., Rong, H. and He, j., 2017. The discovery and significance of Late Triassic marine bivalves in northern Youjiang Basin of Guangxi. Journal of Stratigraphy, 41(3), 273-277.

Xie, T. et al., 2019. New information of fossil bivalves from the luoping biota. Journal of Stratigraphy, 43(4), 411-416.

Xu, G. and Grant, R.E., 1994. Brachiopods near the Permian-Triassic boundary in South China.

Yang, S.-R., Wang, X. and Hao, W., 1986. Early Triassic bivalve assemblage of western Guangxi. Journal of Stratigraphy, 10(2), 88-97.

Yang, T., 2015. The bivalve fauna from deep-water facies of south China during the Permian–Triassic interval. China University of Geosciences. 1-120.

Yang, Z. and Xu, G., 1966. Brachiopods of Middle and Upper Triassic from Central Guizhou.

Yang, Z.Y. et al., 1987. Permian–Triassic boundary stratigraphy and fauna of South China. PRC Ministry of Geology and Mineral Resources, Geological Memoirs, 26, 379.

Yanwei, Z., Baoyu, J. and Yazhuo, N., 2014. Study on the upper Triassic-lower Jurassic bivalve biostratigraphy and the Triassic-Jurassic boundary in the Nanling region, south China. Geological Journal of China Universities, 20(4), 570.

Yao, H. et al., 2003. A new genus *Quemocuomegalodon* of megalodontidae from the upper triassic in the source area of the Yangtze River, western China. Acta Palaeontologica Sinica, 42(3), 393-407.

Yao, H., Zhang, R., Pojeta, J., Sha, J. and Wang, J., 2007. Late Triassic megalodontids (Bivalvia) from the headwaters of the Yangtze River, Qinghai province, west China. Journal of Paleontology, 81(6), 1327-1347.

Yin, J. and Grant‐Mackie, J., 2005. Late Triassic-Jurassic bivalves from volcanic sediments of the Lhasa block, Tibet. New Zealand Journal of Geology and Geophysics, 48(3), 555-577.

Yu, S., Zhang, X., Yang, Z., Wang, J. and Wen, Q., 2011. Late permian bivalve fossils in Hami, Xinjiang and their geological signifiences. Xinjiang Geology, 29(1), 43-46.

Zhang, Y., He, W.-H., Shi, G., Zhang, K.-X. and Wu, H.-T., 2015. A new Changhsingian (Late Permian) brachiopod fauna from the Zhongzhai section (South China) part 3: Productida. Alcheringa: An Australasian Journal of Palaeontology, 39(3), 295-314.

Zhang, Z., 1987. Biostratigraphy of the Three Gorges area 4 The Triassic-Jurassic Volume.

Zhongming, Z., Linong, L., Xiaona, Y., Wangqiang, Z. and Wei, L., 1978. Paleontological Atlas of Southwest China.

Zhongming, Z., Linong, L., Xiaona, Y., Wangqiang, Z. and Wei, L., 1979. Paleontological Atlas of Northwest China.

Zhongming, Z., Linong, L., Xiaona, Y., Wangqiang, Z. and Wei, L., 1980. Late Permian coal-bearing strata and paleontological groups in western Guizhou and Eastern Yunnan.

Zhu, L., Ding, M. and Liu, H., 1992. The discovery of ammonoid and bivalve faunas of the lower triassic and their stratigraphic significance in Shatian, Daye County, Hubei Province. Earth Science–Journal of China University of Geosciences, 17(3), 337-344.

Zongjie, F. and Dewei, Y., 1995. Discovery of fossil bivalves from Ealy Permian of Dongfang, Hainan Island with a review on glaciomarine origin of Nanlong diamictites. Acta Palaeontologica Sinica, 34(3), 301-315.
